# Influence of Ambidextrous Learning on Eco-Innovation Performance of Startups: Moderating Effect of Top Management’s Environmental Awareness

**DOI:** 10.3389/fpsyg.2020.01976

**Published:** 2020-08-18

**Authors:** Shi-zheng Huang, Jian-ying Lu, Ka Yin Chau, Hai-liang Zeng

**Affiliations:** ^1^School of Economics and Management, Guangdong University of Petrochemical Technology, Maoming, China; ^2^Guangxi University of Finance and Economics, Nanning, China; ^3^Faculty of Business, City University of Macau, Macau, China; ^4^Faculty of International Tourism and Management, City University of Macau, Macau, China

**Keywords:** ambidextrous learning, green innovation, top management’s environmental awareness, eco-innovation performance, startups

## Abstract

Ecological innovation is an inevitable trend for firms to enhance competitiveness and sustainably operate in the context of green economy. The previous literature has rarely discussed the influence of ambidextrous learning on the eco-innovation performance of startups and ignored the moderating effect of top management’s environmental awareness from the perspective of microscopic psychology. We have conducted a questionnaire survey on 212 firms established within 4 years in the Pearl River Delta of China, using the structure mode and the PROCESS by Hayes (2013) to analyze the influence of ambidextrous learning, such as exploratory learning and exploitative learning, by startups on eco-innovation performance and verify the moderating effect of top management’s environmental awareness. The results show that exploratory learning and exploitative learning have a positive and significant influence on eco-innovation performance, indicating that the organizational learning of startups is conducive to improving eco-innovation performance; under the moderating effect of top management’s environmental awareness, the influence of exploratory learning and exploitative learning on eco-innovation performance may differ. The results also show that in the process of organizing ambidextrous learning, startups should help raise the environmental awareness of top management to improve the eco-innovation performance, thus providing guidance for startups to carry out eco-innovation activities and for local governments to make decisions on green economy.

## Introduction

Green economy has become a new economic development mode for countries around the world to cope with global climate change, food safety, health and disease prevention, and economic recession. Ecological innovation is also a key factor in stimulating the development of green economy (Nidumolu et al., 2009; Orsato, 2009; OECD, 2014; Cai and Li, 2018). Ecological innovation and green technology are developing rapidly in Europe. As the scale of eco-industry gradually expands, the turnover of eco-industry is nearly EUR 2 trillion, which promotes employment and economic growth among European Union (EU) countries (OECD, 2011; Berrone et al., 2013; Costantini et al., 2017). The “Made in China 2025” plan includes “green development, cyclic development, and low-carbon development” as one of the main development directions of China. The national and local governments have successively issued a few regulations to encourage and guide green and low carbon development and accelerate the development of green technology innovation and ecological industrialization. With the increase in environmental awareness of consumers, green products and green consumption are increasingly favored (Burki and Dahlstrom, 2017; Wang et al., 2018), and the top management of firms also realizes that there may be a balance between corporate development and environmental protection and ecological innovation can also form a new niche.

Ecological innovation is an inevitable trend for firms to enhance competitiveness and sustainably operate in the context of “green economy” (Christensen, 1995; Kriecher and Ziesemer, 2009; He et al., 2019; Liu et al., 2019; Lopes Santos et al., 2019). Based on the development of innovation management theory, ecological innovation has gone through the stages of technological innovation and green innovation. Driven at multiple levels, including society, system, organization and individual, ecological innovation involves multiple dimensions such as product, process, organization, marketing, society, system, etc. (OECD, 2010; O’Brien et al., 2013; Lin et al., 2014; Hojnik et al., 2018; Huang et al., 2018; Salim et al., 2018). The organizational learning theory, based on cognitive psychology, believes that organizations can achieve organizational objectives by acquiring and further understanding better knowledge and improving their actions (Argyris and Schon, 1978; Fiol and Lyles, 1985; Witkin, 1965). Organizational learning is not only creative learning, but also long-term cultivation and the process of shaping corporate value (Kharabsheh et al., 2015; Sikora et al., 2016; Huang and Li, 2017; Yuan et al., 2019; Wu et al., 2020). Therefore, organizational learning is a prerequisite for the continuous change and innovation of organizations and also plays a vital role in influencing eco-innovation performance during the process of ecological innovation. March (1991) divides ambidextrous learning of organizations into exploratory learning and exploitative learning. Exploratory learning allows new knowledge to be obtained from external sources, so that startups can more quickly perceive and grasp external opportunities and make better use of the acquired knowledge to reorganize corporate resources and better integrate and create knowledge. Exploitative learning can help startups have a deeper understanding of external environment and their resources and improve their ability to sense opportunities and threats (Baker and Sinkula, 1999; Lichtenthaler, 2009; Yuan et al., 2019).

The upper echelons theory believes that the top management, affected in decision-making by their personal experiences, cognitive models, values, environmental attitudes, and other characteristics, may perceive the external environment, identify opportunities and threats, and make scientific decisions according to their characteristics (Drucker, 1985; Hanbrick and Mason, 1984; Hambrick, 2007; Wu et al., 2018; Wu and Wu, 2019). The top management plays an important role in selecting corporate strategies (Child, 1997). As the market environment changes and the environmental protection pressure in the business environment increases, the top management also realizes the need for ecological innovation through green products and green technologies. From the perspective of cognitive psychology, the influence of internal and external factors on green innovation mainly depends on the perception and interpretation of environment by the top management. The stronger the environmental awareness of the top management is, the more they tend to identify the potential benefits and new niche of green innovation (Christensen, 1995; Drucker, 1985; He et al., 2019; Porter, 1995; Wu and Wu, 2019). The stronger the environmental awareness is, the more the top management will use organizational learning to perceive and grasp external opportunities, acquire new external knowledge, and integrate and create knowledge. Therefore, analyzing the relationship between cognition and behavior from the microscopic psychology perspective of the top management is conducive to a better and more comprehensive understanding of eco-innovation performance.

However, the previous literature has rarely discussed the influence of ambidextrous learning on the eco-innovation performance of startups and ignored the moderating effect of top management’s environmental awareness from the perspective of microscopic psychology. Because of the characteristics of startups, the influence of explanatory learning and exploitative learning on eco-innovation performance may vary during their process of growth and development. Therefore, this article, based on cognitive psychology, constructs a “cognition–behavior performance” research model according to the organizational learning theory and the upper echelons theory to explore the influence of ambidextrous learning on the eco-innovation performance of startups and to verify the moderating effect of the top management’s environmental awareness, validating the moderating effect of the top management’s environmental awareness on ambidextrous learning and eco-innovation performance based on the upper echelons theory, exploring different influences on startups in internal and external environments, enriching the researches on action mechanism of ambidextrous learning and eco-innovation performance, and providing profound guidance for improving the eco-innovation performance of startups.

## Theoretical Basis and Hypothesis

### Ambidextrous Learning and Eco-Innovation Performance

Based on the theoretical basis of organizational learning, March (1991) first proposed the concept of ambidextrous learning during his research of organizational adaptation and development and divided ambidextrous learning of organizations into exploitative learning and exploratory learning. Exploratory learning is essentially a type of trial-and-error and continuously verified learning behavior, emphasizing the in-depth excavation of internal and external resources of organizations, and constant trial, adventure, and innovation. Exploitative learning is essentially a learning behavior that summarizes and refines existing knowledge. Exploitative learning is conducive to organizations’ deep understanding of acquired knowledge and is of great significance to the survival of organizations. For startups, ambidextrous learning is more an entrepreneurial learning behavior (exploratory learning and exploitative learning). Ambidextrous learning, generally related to entrepreneurial activities, allows startups to continuously create and accumulate knowledge during their growth and development process. Ambidextrous learning runs through the entire life cycle of startups.

Eco-innovation performance is generated by organizations adapting to environment changes during the development of innovation management, which essentially requires organizations to integrate internal and external resources and technologies, invest in R&D and green technology, reduce environmental pollution and improve environmental performance, save resources and improve socioeconomic results. Therefore, eco-innovation performance includes R&D investment, corporate performance, economic benefits, environmental effects, and so on. Nehrt (1998) pointed out that, according to the perspective of resource-based view, the ways to reduce pollution include product design, equipment, process, raw material recycling, and so on, and organizations should reduce costs and prevent pollution while creating market demand. The development of eco-innovation performance requires organizations to continuously accumulate and acquire knowledge. To a certain extent, the formation and development of eco-innovation performance depend on organizations’ acquisition and accumulation of knowledge in this process. This shows that learning is the main mechanism for the creation and development of eco-innovation performance, and trial and error, improvisation, and imitation of organizational learning can more effectively improve organizations’ eco-innovation performance.

Compared with mature firms, the ambidextrous learning of startups has a more significant influence on organizational resources and capabilities. First, the exploration and use of external knowledge are essential for startups to upgrade and update existing resources and rebuild new resources; second, the “new” characteristics of startups reflect that existing knowledge of startups may not be sufficient to satisfy current developments, and startups must transform the information and technologies acquired through ambidextrous learning into organizational resources (He et al., 2018; Wu et al., 2018; Wu and Wu, 2019). Exploratory learning and exploitative learning, as two types of organizational learning, play an important role in improving the green innovation performance of organizations (Easterby-Smith and Prieto, 2008; He et al., 2019). Exploratory learning can improve the green innovation performance of startups by allowing startups to acquire new entrepreneurial knowledge from external sources and helping startups to perceive opportunities and enhance their creation abilities, so that startups can more quickly perceive and grasp external opportunities and make better use of the acquired knowledge to reorganize corporate resources and better integrate and create knowledge (Lichtenthaler, 2009). In this process, organizations will better adapt to environment changes through repeated trial and error and correction.

Exploitative learning can improve the green innovation performance of startups by emphasizing the upgrading of existing knowledge of startups and helping startups respond to organizational changes timely and improve the green innovation performance through expanding the content and depth of the knowledge resources of startups (Liao et al., 2009; Bi et al., 2015). Lin and Chen (2017) focused on knowledge resources and capabilities and found that the sharing of green knowledge can improve the green innovation performance. Exploitative learning can help startups have a deeper understanding of external environment and their resources and improve their ability to sense opportunities and threats. In addition, exploitative learning emphasizes the further refinement of knowledge and strengthens the understanding of organizational practices and processes, thus helping startups better adapt to environmental changes and improve their eco-innovation performance. Therefore, this article believes that explanatory learning and exploitative learning are of great importance to eco-innovation performance during the development process of startups and puts forward the following hypotheses:

*Hypothesis 1*: Exploratory learning of startups has a positive influence on eco-innovation performance.

*Hypothesis 2*: Exploitative learning of startups has a positive influence on eco-innovation performance.

### Interactive Ambidextrous Learning and Eco-Innovation Performance

Existing researches have different perspectives on the opposite and complementary relation between exploratory learning and exploitative learning. The view for opposite relation believes that exploratory learning and exploitative learning will compete for the resources of organizations, and organizations have a certain inertia and dependence on exploitative learning or exploratory learning. Both types of learning activities require different organizational mechanisms (March, 2006). The simultaneous implementation of exploratory learning and exploitative learning may have a negative influence on corporate performance, so organizations can carry out only one type of learning activity. The view for complementary relation believes that activities of exploratory learning and exploitative learning can be carried out simultaneously, and organizations can control both types of learning activities at the same time (Zollo and Winter, 2002). In recent years, more researches have shown that exploratory learning and exploitative learning are complementary rather than opposite.

Exploratory learning essentially means the discovery and acquisition of new external knowledge, which is vital to the future development of organizations. Exploitative learning means the upgrade and transformation of existing entrepreneurial knowledge of organizations and the improvement of current performance of organizations. Exploratory learning supplements startups with new entrepreneurial knowledge and increases their existing knowledge that in turn promotes the acquisition of new knowledge. Therefore, the existing and new knowledge jointly improve the eco-innovation performance of startups. As the organizational environment becomes more turbulent, the knowledge required by organizations also keeps changing, and the perspective for interactive ambidextrous learning is increasingly recognized by the academic community. Organizations realize their creation and accumulation of knowledge through exploratory learning and exploitative learning. The cyclic development of knowledge will be formed when new knowledge acquired from external sources is transformed into existing knowledge and implicit knowledge is transformed into explicit knowledge (Nonaka et al., 2000; Yuan et al., 2019; Wu et al., 2020). Therefore, the interactive ambidextrous learning of organizations is a cyclic process. Exploitative learning cannot occur without exploratory learning, and the use of existing resources by organizations must be based on previous exploratory learning. In addition, the startups committed to exploration and innovation must adopt exploitative learning. Providing stable financial supports for high-risk exploratory learning can also promote startups to explore new entrepreneurial knowledge more effectively. Exploratory and exploitative learning can promote the incremental and disruptive innovation of startups (Lin, 2015). It further shows that interactive ambidextrous learning is more important to the eco-innovation performance, and ignoring any of them may have a certain influence on the corporate development. This article proposes that interactive ambidextrous learning has a positive influence on the eco-innovation performance and puts forward Hypothesis 3:

*Hypothesis 3*: Interactive ambidextrous learning of startups has a positive influence on eco-innovation performance.

### Moderating Effect of Top Management’s Environmental Awareness

The upper echelons theory believes that the top management, affected by their personal experiences, cognitive models, values, and environmental attitudes (Hambrick, 2007), is the core of business management and also a key role in corporate strategic decisions (Child, 1997). The cognitive psychology believes that consciousness is the response of human brain to the objective existence in environment and is reflected in people’s awareness and attention to the outside world and their own environment (Hanbrick and Mason, 1984; Witkin, 1965), and environmental awareness, which is a concrete embodiment of cognitive models, embodies individuals’ perception and behavioral tendency toward environmental issues. The influence of internal and external factors on green innovation mainly depends on the perception and interpretation of environment by the top management, whereas the decision to carry out eco-innovation activities depends on the environmental awareness of the top management. The previous literature indicates that the top management knows that environmental opportunities may become an important source of increases in actual income. The stronger the environmental awareness is, the more the top management tends to identify the potential benefits and market opportunities of green innovation (Drucker, 1985; Christensen, 1995; Porter, 1995; Berrone et al., 2013; Feng and Chen, 2018; Wang and Zhang, 2018).

With a stronger environmental awareness, the top management will also have a strong sense of responsibility for green innovation and is willing to invest more resources and efforts into the green innovation field. With limited resources, the return on investment is the primary consideration for organizations’ project investment. Given the large amount of resource input for green innovation, high market risks, and significant R&D uncertainty, only when the top management incorporates green innovation into the scope of corporate responsibility will it invest more resources from a strategic height (Kharabsheh et al., 2015; Zhang and Walton, 2016; Huang and Li, 2017; Zhang and Lv, 2018). The top management with a stronger environmental awareness holds an open and supportive attitude toward green innovation, is good at exploratory learning, encodes and integrates the acquired information with corporate resources, absorbs internal and external knowledge of organizations and applies it to green innovation, and proactively responds to environmental issues. The top management with a strong environmental awareness can utilize the exploitative learning to help organizations identify external market opportunities and reasonably allocate their resources and capabilities. Sharma (2000) analyzes the process of strategic selection by organizations from the perspective of opportunities and threats perceived by the top management. He believes that the top management who considers environmental issues as opportunities will tend to select proactive environmental strategies. Burki and Dahlstrom (2017) pointed out that the top management’s attitudes and commitments to the environment affect green innovation, which in turn may be conducive to establishing a good cooperation environment.

The previous literature indicates that organizational learning behavior can help organizations be more sensitive to external opportunities, thereby improving their competitive advantages. However, learning activities alone cannot help organizations gain real competitive advantage. The environmental awareness of the top management, as a type of cognitive consciousness, has a moderating effect as it can more clearly reflect the value effect of learning activities. That is to say, the development of exploratory learning and exploitative learning activities will be affected by the environmental awareness of the top management. A stronger environmental awareness of the top management will have a more significant influence on organizational ambidextrous learning and eco-innovation performance. Therefore, this article believes that the environmental awareness of the top management has a moderating effect on the ambidextrous learning (including learning and exploitative learning) of startups and eco-innovation performance and puts forward the following hypotheses:

*Hypothesis 4a*: With a stronger environmental awareness of the top management, the exploratory learning will have a more significant positive influence on eco-innovation performance.

*Hypothesis 4b*: With a stronger environmental awareness of the top management, the exploitative learning will have a more significant positive influence on eco-innovation performance.

*Hypothesis 4c*: With a stronger environmental awareness of the top management, the interactive ambidextrous learning will have a more significant positive influence on eco-innovation performance.

**FIGURE 1:**
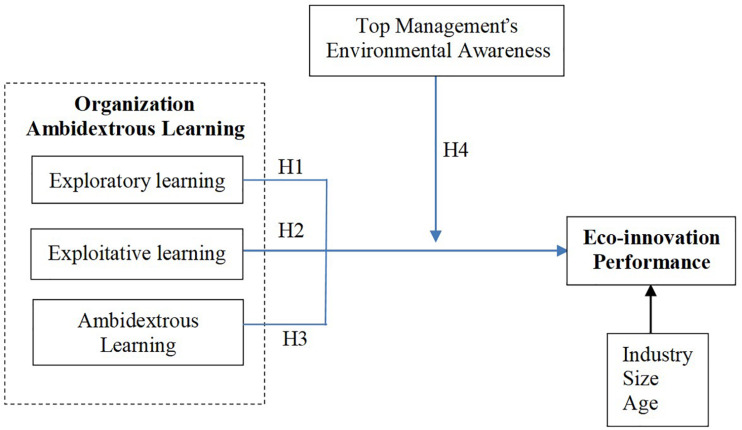
Research framework.

In conclusion, this article, based on organizational learning theory, upper echelons theory, and cognitive psychology, explores the influence of ambidextrous learning on the eco-innovation performance of startups and verifies the moderating effect of the top management’s environmental awareness. Its research framework is shown in Figure 1.

## Materials and Methods

### Sample and Data Collection

The data of this article came from the startups established within 4 years in the Pearl River Delta of China. Sampling targets were the startups defined by Global Entrepreneurship Monitor that were established within 42 months and in the period of establishment or growth. In this survey of major respondents of senior management, with the help of Guangdong SME Bureau and Shenzhen SME Service Bureau, we conducted a written questionnaire random sampling survey on the trainees participating in the training program for SME senior management. A total of 500 questionnaires were sent out, of which 268 questionnaires were collected, accounting for 53.6%. In addition to 56 questionnaires with incomplete information, there were 212 valid questionnaires, accounting for 79.10%. Samples indicated that among the industries of sampled firms, manufacturing accounted for 38.68%, information transmission, software and information technology services industry accounted for 22.64%, financial industry accounted for 16.04%, logistics industry accounted for 8.96%, retail industry accounted for 5.66%, and construction industry accounted for 8.02%; in terms of firm nature, state-owned firms accounted for 5.66%, private firms accounted for 89.15%, and foreign-invested firms accounted for 5.19%; in terms of firm scale, firms with 1 to 10 people accounted for 28.77%, firms with 11 to 50 people accounted for 20.28%, firms with 51 to 100 people accounted for 46.70%, and firms with 101 people or more accounted for 4.25%; the sampled firms had an average age of 1.6 years, of which 10.85% were established within 1 year, 40.57% within 1 to 2 years, 31.13% within 2 to 3 years, and 17.45% within 3 to 4 years; the targets of questionnaire survey mainly include senior and middle management personnel of firms, of which 78% were males and 22% were females, 8.49% were doctors, 50.63% were masters, 30.50% were undergraduates, and 10.38% were high school students or below; 20.28% were chairman, 50.00% were general (vice general) manager, 25.47% were department manager, and 4.25% were other personnel. The average length of service of current employees was 1.8 years, and their average age was 31 years. The results are shown in Table 1.

**TABLE 1:** Distribution of the sampled firms.

	*N* = 212	Percentage
**Industry**		
Manufacturing	82	38.68%
Information transmission, software, and information technology services	48	22.64%
Finance	34	16.04%
Logistics	19	8.96%
Retail	12	5.66%
Construction	17	8.02%
**Firm nature**		
State-owned	12	5.66%
Private	189	89.15%
Foreign-invested	11	5.19%
**Firm scale**		
1–10	61	28.77%
11–50	43	20.28%
51–100	99	46.70%
≥ 101	9	4.25%
**Firm age**		
Within 1 year	23	10.85%
1–2 years	86	40.57%
2–3 years	66	31.13%
3–4 years	37	17.45%

### Measures

The questionnaire questions in this research were mainly designed according to previous theoretical basis and related literature. To ensure the preciseness of questionnaire, the first edition of questionnaire should be provided with guidance from scholars, professors, and expert practitioners. Through multiple amendments of items and grammar, the questionnaire was pretested by 30 selected startups, and the pretest statistical analysis ensured the accuracy, fitness, and convenience of the questionnaire items before reamendment and finalization of questionnaire. The questionnaire used the 7-point Likert scale. The higher the number is, the more the respondents agree with the description (1 means strongly disagree, 3 means agree, and 7 means strongly agree). The construct variables of questionnaire mainly included eco-innovation performance, exploratory learning and exploitative learning under organizational ambidextrous learning, and the top management’s environmental awareness. The operational definitions of variables in the research framework and the basis for research scale are explained below.

### Eco-Innovation Performance

Eco-innovation performance was a dependent variable, which was further extended, revised and integrated into this research based on the EU eco-innovation performance assessment indicators and the green innovation performance scale (Cai and Li, 2018; He et al., 2019; OECD, 2014; Ryszko, 2017), along with the Chinese eco-innovation models. There are five items in total, including “eco-innovation investment,” “eco-innovation activities,” “eco-innovation output,” “effect of resource conservation,” and “socioeconomic results.” According to the results, the Cronbach α of this scale is 0.92, indicating a good reliability.

### Organizational Ambidextrous Learning

Organizational ambidextrous learning is an independent variable, including exploratory and exploitative learning. This scale is based on the scale developed by Atuahene-Gima and Murray (2007), He et al. (2019), Su et al. (2011). Exploratory learning mainly includes four items, including “Acquiring new entrepreneurial knowledge and skills for firms,” “Acquiring brand-new product development technologies or development processes for firms to develop new entrepreneurial opportunities,” “Using new management and organizational skills to develop entrepreneurial opportunities,” and “Obtaining new knowledge and skills for investment and development of new technologies and allocation of R&D functions to integrate entrepreneurial resources.” According to the results, the Cronbach α of this scale is 0.78, indicating a good reliability.

Exploitative learning mainly included five items, including “Upgrading of existing entrepreneurial knowledge and skills by firms in the field of products and technologies,” “Strengthening the upgrading of existing technologies to improve the efficiency of opportunity exploitation,” and “Actively seeking solutions for customer problems and improving the utilization efficiency of existing entrepreneurial resources,” “Further improving entrepreneurial skills in the development of new products with certain experience,” and “Accumulating entrepreneurial knowledge and experience to improve the efficiency of entrepreneurial activities.” According to the results, the Cronbach α of this scale is 0.86, indicating a good reliability.

### Top Management’s Environmental Awareness

Top management’s environmental awareness is a moderating variable. This scale is based on the researches of Eiadat et al. (2008), Gadenne et al. (2009), and Peng and Wei (2015) and mainly includes three items, including “The top management attaches importance to the influence of environmental protection regulations on firms,” “The top management attaches importance to the adverse influence of production and operation activities of firms on environment,” and “The top management attaches importance to the understanding and mastery of environmental protection measures.” According to the results, the Cronbach α of this scale is 0.87, indicating a good reliability.

### Control Variables

In this research, industry, firm nature, firm scale, and firm age are selected as control variables. Because industry, firm nature, firm scale, and firm age of startups have different influences on corporate performance (Chen et al., 2012; He et al., 2019), control variables can have an influence on environmental awareness of the top management, ambidextrous learning, and eco-innovation performance.

## Analyses and Results

### Average Verification

SPSS 23.0 statistical analysis software was used in this research to perform descriptive statistical analysis of sample data. The mean, standard deviation, and correlation coefficient of each variable are shown in Table 2. Table 2 shows that the correlation coefficients of eco-innovation performance, exploratory learning, exploitative learning, and environmental awareness of top management were between 0.51 and 0.78, and all reached a significant level, indicating that there is a moderately positive correlation between construct variables and eco-innovation performance. Given that each questionnaire is filled by one survey subject, the data source may have a common method bias. Harman single factor method was used to solve the common method bias problem. The analysis shows that in the case of no rotation the first factor explains 38.50% of variance, which did not account for the majority, indicating that the common method bias in this research would not affect the research results.

**TABLE 2:** Basic descriptive statistics of the correlation coefficients.

Variable	Mean	Std	1	2	3	4	5	6	7
1. Eco-innovation performance	3.93	0.56	1						
2. Exploratory learning	3.76	0.63	0.64**	1					
3. Exploitative learning	3.63	0.82	0.51**	0.63**	1				
4. Top management’s environmental awareness	3.86	0.51	0.72**	0.78**	0.60**	1			
5. Industry	3.85	2.86	0.12	0.15	–0.08	–0.10	1		
6. Size	3.54	1.02	−0.14**	−0.12**	−0.17**	−0.21**	0.01	1	
7. Age	3.50	0.83	–0.11	–0.09	–0.12	–0.13	−0.10	−0.17*	1*
CR			0.88	0.78	0.85	0.92			
AVR			0.68	0.64	0.67	0.63			

*N* = 212. *p < 0.05, **p < 0.01 (two-tailed test).

### Confirmatory Factor Analysis

In this research, Mplus 7.0 statistical analysis software was used to verify the reliability, convergent validity, and discriminant validity of questionnaire scale with confirmatory factor analysis. The results are shown in Table 2. The Cronbach α’s of all research variables are higher than 0.7. The composite reliability was higher than 0.7, the average variance extracted was up to 0.5, and the convergent validity reached the recommended standard of related scholars (Anderson and Gerbing, 1988; Fornell and Larcker, 1981). If the result of confidence interval test shows that the upper and lower limits of correlation coefficient between construct variables do not contain 1 after adding or subtracting two standard errors, then the questionnaire has good discriminant validity (Bagozzi and Yi, 1988). CFA results show that χ^2^/df = 2.06 < 3, *p* < 0.001; SRMR = 0.05 < 0.08; CFI = 0.93 > 0.90; IFI = 0.91 > 0.90; RMSEA = 0.06 < 0.08, indicating that the questionnaire has good reliability and validity, and the scale used has good measurement qualities (Hu and Bentler, 1999).

### Hypothesis Testing

In this research, the statistical analysis software PROCESS proposed by SPSS 23.0 and Hayes (2013) was used to carry out hypothesis verification on sample data. The results are shown in Table 3. Model 1 was to verify the control variables, Model 2 in Table 3 was used to verify major variables. The results show that exploratory learning has a significant positive influence on eco-innovation performance (β = 0.62, *p* < 0.001), so hypothesis 1 is supported; exploitative learning has a significant positive influence on eco-innovation performance (β = 0.43, *p* < 0.001), so hypothesis 2 is supported; the environmental awareness of top management has a significant positive influence on eco-innovation performance (β = 0.48, *p* < 0.001). Model 3 was used to verify the interaction. The results show that the interaction between exploratory learning and exploitative learning has no significant influence on eco-innovation performance (β = 0.08, *p* > 0.05), and hypothesis 3 is not supported. The interaction between environmental awareness of top management and exploratory learning has no significant positive influence on eco-innovation performance (β = 0.16, *p* > 0.05), and hypothesis 4a is not supported, indicating that the environmental awareness of top management has no moderating effect on the relationship between exploratory learning and eco-innovation performance; the interaction between environmental awareness of top management and exploitative learning has no significant positive influence on eco-innovation performance (β = 0.23, *p* > 0.05), and hypothesis 4b is not supported, indicating that environmental awareness of top management has no moderating effect on the relationship between exploitative learning and eco-innovation performance.

**TABLE 3:** Results of moderated multiple regression analysis.

Variable	Eco-innovation performance			
	Model 1	Model 2	Model 3	Model 4
**Control**				
Industry	−0.10	−0.02	−0.02	-0.08
Size	−0.21*	−0.03	−0.02	0.01
Age	0.09	0.01	0.01	−0.01
**Main effects**				
Exploratory learning		0.62***	0.58***	0.32***
Exploitative learning		0.43***	0.37***	0.24***
Top management’s environmental awareness		0.48***	0.43***	0.38***
**Two-way interactions**				
Exploratory learning exploitative learning			0.08	0.04
Exploratory learning top management’s environmental awareness			0.16	0.11
Exploitative learning top management’s environmental awareness			0.23	0.18
**Three-way interaction**				
Ambidextrous learning (exploratory learning exploitative learning) top management’s environmental awareness				0.23**
*R*^2^	0.05	0.63	0.60	0.75
Δ*R*^2^	0.04	0.61	0.58	0.72
*F*	3.68*	58.60***	51.43***	56.48***

*N* = 212, *p < 0.05, **p < 0.01, ***p < 0.001.

**FIGURE 2:**
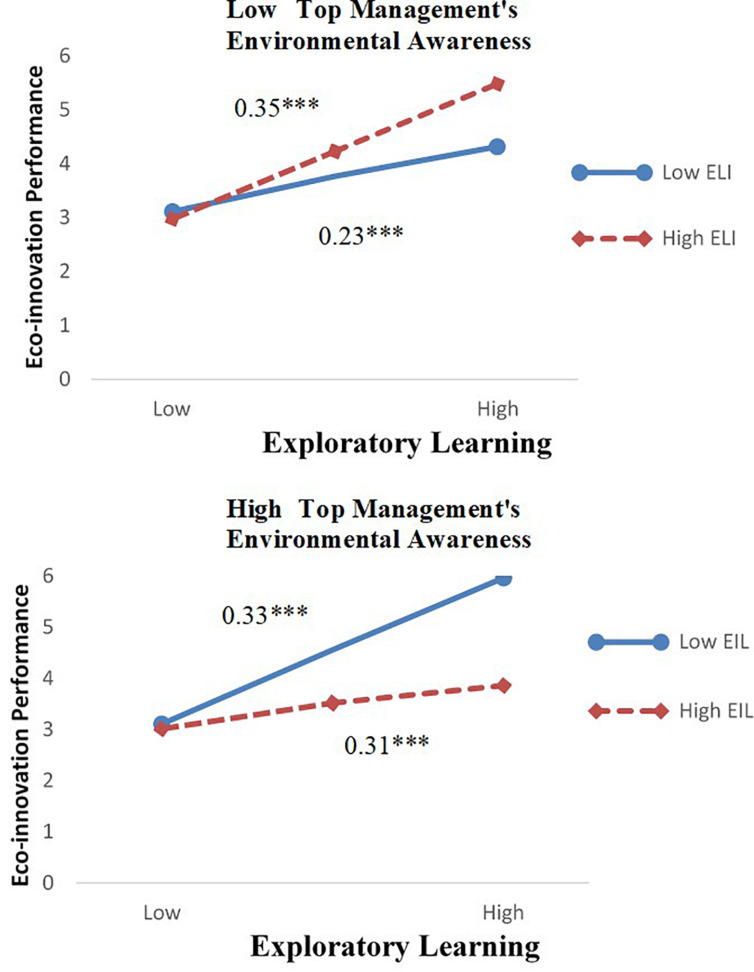
Three-way interaction among ambidextrous learning and top management’s environmental awareness. *N* = 212, *p < 0.05, **p < 0.01, ***p < 0.001.

Model 4 in Table 3 was used to verify three interactions by dividing the top management’s environmental awareness and exploitative learning into low- and high-level types. The results show that the three interactions among the top management’s environmental awareness and exploratory learning and exploitative learning have a significant influence on eco-innovation performance (β = 0.23, *p* < 0.01). When the top management’s environmental awareness is weak, the interaction between exploratory learning and low-level exploitative learning has a significant positive influence on eco-innovation performance (β = 0.23, *p* < 0.001), and the interaction between exploratory learning and high-level exploitative learning has a significant positive influence on eco-innovation performance (β = 0.35, *p* < 0.001); similarly, when the top management’s environmental awareness is strong, the interaction between exploratory learning and low-level exploitative learning has a significant positive influence on eco-innovation performance (β = 0.33, *p* < 0.001), and the interaction between exploratory learning and high-level exploitative learning has a significant positive influence on eco-innovation performance (β = 0.31, *p* < 0.001). The analysis results are shown in Figure 2. Figure 2 indicates that when the top management’s environmental awareness is weak, the coexistence of exploratory learning and high-level exploitative learning on eco-innovation performance has a stronger influence on eco-innovation performance than the coexistence of exploratory learning and low-level exploitative learning, indicating that startups should adopt exploitative innovation to quickly meet market demand by imitating and duplicating products, when the top management has a weak environmental awareness. When the top management’s environmental awareness is strong, the coexistence of exploratory learning and low-level exploitative learning on eco-innovation performance has a stronger influence on eco-innovation performance than the coexistence of exploratory learning and high-level exploitative learning. It shows that startups should adopt exploratory innovation and lead the market by developing new products and inventions to meet the needs of customers, when the top management has a strong environmental awareness.

## Discussion

### Research Conclusion

This article, through empirical analysis, explores the relationship between ambidextrous learning by startups and eco-innovation performance and verifies the moderating effect of the top management’s environmental awareness. The results show that exploratory learning and exploitative learning can respectively promote the improvement of eco-innovation performance, but interactive ambidextrous learning has no significant influence on eco-innovation performance. This shows that both types of entrepreneurial learning activities can improve eco-innovation performance, but this complementary effect may be affected by the balance between two types of learning and other factors.

The environmental awareness of the top management has no moderating effect on the relationship among exploratory learning, exploitative learning, and eco-innovation performance, but has a significant moderating effect on the relationship between interactive ambidextrous learning and eco-innovation performance. It shows that the influence of interactive ambidextrous learning on eco-innovation performance is affected by the environmental awareness of the top management, which plays an important role in startups. When the top management’s environmental awareness is weak, exploratory learning and high-level exploitative learning have a stronger influence on eco-innovation performance than low-level exploitative learning, mainly because when the top management’s environmental awareness is weak, it is difficult for startups to acutely sense external opportunities, and the use, upgrade, or imitation of existing knowledge, and the learning of other people’s experiences can largely meet their own needs for development and capability improvement; when the top management’s environmental awareness is strong, the top management is not satisfied with the use of existing resources, more willing to actively obtain new external information, and dares to take risks and try new things, so organizations are more focused on the pursuit of new knowledge, and the combination of exploratory learning and low-level exploitative learning is more conducive to the improvement of eco-innovation performance.

### Theoretical Implications

This article, based on cognitive psychology, constructs a “cognition–behavior performance” research model according to the organizational learning theory and the upper echelons theory, thus providing theoretical support for how startups can improve their eco-innovation performance. Its main theoretical contributions include the following: First, applying the organizational learning theory to entrepreneurial cases, exploring the influence of exploratory and exploitative learning on the eco-innovation performance, and indicating that ambidextrous learning by startups are essential for ecological innovation (Lichtenthaler, 2009; March, 2006; O’Brien et al., 2013); second, in startups, top management’s environmental awareness may regulate the improvement in eco-innovation performance by ambidextrous learning in various degrees. The influence of organizational ambidextrous learning on eco-innovation performance is also different. It shows that the top management’s environmental awareness plays an important role in the implementation of green innovation activities by startups (Hanbrick and Mason, 1984; Hambrick, 2007). Startups should continuously improve the top management’s environmental awareness, timely sense the changes in external environment, and take measures to promote their development.

### Managerial Implications

The ambidextrous learning of startups can improve their eco-innovation performance. Ecological innovation is a key factor in stimulating the development of green economy (Nidumolu et al., 2009; Orsato, 2009; OECD, 2014) and is also an inevitable trend for firms to enhance competitiveness and sustainably operate (Christensen, 1995; He et al., 2019). With the increase in environmental awareness of consumers, green products and green consumption are increasingly favored (Burki and Dahlstrom, 2017; Wang et al., 2018); startups should carry out ambidextrous learning, perceive and grasp external opportunities, and make better use of the acquired knowledge to reorganize corporate resources, better integrate knowledge, and create new niche (Christensen, 1995; Drucker, 1985; Lichtenthaler, 2009; March, 2006). Startups should adopt exploitative innovation to quickly meet market demand and achieve incremental innovation by imitating and duplicating products, when the top management has a weak environmental awareness. Startups should adopt exploratory innovation and lead the market to achieve disruptive innovation by developing new products and inventions to meet the needs of customers, when the top management has a strong environmental awareness (Lin, 2015). Exploratory learning and exploitative learning should also interact with each other to enhance the innovation capability of startups through learning and imitation, so that startups can realize independent innovation and form a virtuous circle of innovation.

Promote the top management’s environmental awareness. The top management’s environmental awareness plays an important role in carrying out green innovation activities among startups (Hanbrick and Mason, 1984; Hambrick, 2007). Environmental awareness comes from the identification of opportunities, so startups should create a good green atmosphere, give full play to the “decisive role of markets in resource allocation,” and guide consumers to favor green products and green consumption (Burki and Dahlstrom, 2017; Wang et al., 2018). Startups should carry out green innovation activities, invest in research and development of green products, promote clean production and environmental management systems, and build ecological supply chains, thereby forming a virtuous circle dominated by green competitive advantages. Environmental awareness comes from the responsibility sense of top management. Startups should cultivate the top management’s environmental awareness through trainings, lectures, new media platforms, and so on; guide the top management to address the demands of stakeholders, such as governments, communities, and non-profit organizations, and actively consider environmental issues as the responsibilities of top management; make commitments for resources and efforts; and then carry out green innovation to improve their eco-innovation performance.

## Limitations and Future Study Directions

Future researches can introduce more entrepreneurial contextual variables, for example, by studying the influences on eco-innovation performance of startups from an institutional or individual perspective. In addition, given the limited quantity of samples, some biases may exist in the research results, so the diversity of samples is suggested to be increased.

## Data Availability Statement

The original contributions presented in this study are included in this article/supplementary material. Further inquiries can be directed to the corresponding authors.

## Ethics Statement

This study was carried out in accordance with the recommendations of ethics committee of Guangdong University of Petrochemical Technology with written informed consent from all subjects in accordance with the Declaration of Helsinki. The protocol was approved by the ethics committee of Guangdong University of Petrochemical Technology.

## Author Contributions

SH led the research design, data analysis, and drafted this manuscript. JL guided the research design and revised the manuscript substantially. KC and HZ made contributions in data analysis and manuscript revision. All authors approved the final version.

## Conflict of Interest

The authors declare that the research was conducted in the absence of any commercial or financial relationships that could be construed as a potential conflict of interest.
